# Single-cell sequencing decodes the secrets of the RAP phenomenon of corticotomy

**DOI:** 10.3389/fimmu.2024.1397727

**Published:** 2024-10-01

**Authors:** Zhibo Fan, Shenghong Li, Liping You, Yuxin Lan, Yutong Zhong, Yuefan Ma, Jie Xu, Xiaomei Xu

**Affiliations:** ^1^ Department of Orthodontics, The Affiliated Stomatological Hospital of Southwest Medical University, Luzhou, China; ^2^ Oral & Maxillofacial Reconstruction and Regeneration of Luzhou Key Laboratory, The Affiliated Stomatological Hospital of Southwest Medical University, Luzhou, China

**Keywords:** orthodontic tooth movement, orthodontic(s), single-cell sequencing, bone remodeling/regeneration, osteoclast(s)

## Abstract

**Introduction:**

Corticotomy-assisted tooth movement is commonly performed in clinics, however, its time-limited efficacy and the fear of surgery among patients significantly limit its clinical application. Hence, researchers have investigated non-invasive methods to accelerate tooth movement. However, the molecular mechanisms underlying corticotomy-assisted tooth movement are not fully understood.

**Methods:**

Micro-CT and TRAP stain were used to tooth movement and bone resorption. Single-cell RNA sequencing was used to study the transcriptome heterogeneity of macrophages after corticotomy. Transmission electron microscopy and iron ion detection was used to evaluate ferroptosis and iron metabolism. In addition, we carried out immunohistochemistry, quantitative real-time and flow cytometry verify the effect of iron on macrophage polarization.

**Results:**

Single-cell RNA sequencing of digested alveolar bone identified a significant increase in iron metabolism-related genes post-corticotomy. Macrophages play a central role in this field. Following the dimensionality reduction of macrophages, we revealed a new developmental state via pseudotime analysis post-corticotomy. SCENIC analysis revealed that Atf3 is a key transcription factor influencing this new state. We found that Atf3+ macrophages were closely associated with osteoclasts. Moreover, cell chat revealed an increase in cellular communication between Atf3+ macrophages and other cell types after corticotomy.

**Discussion:**

These findings suggested that Atf3+ macrophages might play a key role in corticotomy-accelerated tooth movement, thus providing potential targets for drug development.

## 1 Introduction

Orthodontic tooth movement depends on the bone remodeling process, but orthodontic treatment is time-consuming due to the slow process of mechanical force-induced bone remodeling. The long duration of orthodontic treatment is not only stressful but also increases the risk of caries, periodontal disease, and root resorption (1). The development of corticotomy has considerably improved orthodontic treatment and shortened its duration, leading to its widespread clinical acceptance in the bone remodeling process (2). However, patients do not accept corticotomy (3, 4). Therefore, the molecular mechanism underlying corticotomy needs to be elucidated to accelerate orthodontic tooth movement and develop a new type of long-lasting and non-invasive method to accelerate tooth movement.

Regional acceleration phenomenon (RAP) induced by corticotomy plays an important role in tooth movement acceleration, which is a complex response of mammalian tissues to various noxious stimuli (5). RAP occurs when bone tissue responds to various traumatic stimuli, followed by activation of bone remodeling, resulting in a decrease in local bone density (6); this process is immune environment-dependent (7). The alveolar bone, which is the main bearer of occlusal force, has an active immune microenvironment (8). Traumatic stimuli cause the bone tissue to quickly elicit an immune response, causing a large number of inflammatory factors and chemotactic mediators to be released. These changes induce a large number of monocytes and macrophages to enter, which initiate bone remodeling (9, 10). Macrophages not only participate in the bone remodeling process related to corticotomy (11) but also play a central role in tooth movement (12). The absence of macrophages has a fatal effect on tooth movement (13). Therefore, many researchers have investigated the changes in macrophages after corticotomy and found that after corticotomy, macrophages showed infiltration and an increase in polarization to the M1 type (11), but how macrophages participate in tooth movement acceleration after corticotomy is not clear. As the heterogeneity of macrophages is complex, research on the molecular mechanisms underlying the progress of macrophages is lacking; thus, the role of macrophages in corticotomy is not known.

In this study, we elucidated the mechanism of corticotomy-accelerated tooth movement by simulating corticotomy-accelerated tooth movement surgery in SD rats and performing scRNA-seq. We found that mineralization was lower and osteoclast activity was higher after corticotomy compared to that in the tooth movement group. Bioinformatics analysis suggested an increase in iron metabolism-related genes in monocyte-macrophage subpopulations after corticotomy, and the accumulation of iron post-corticotomy was confirmed by staining tissues. The trajectory analysis showed that macrophages added a new developmental trajectory, in which cells were closely associated with osteoclasts and inflammatory responses and might be regulated by the transcription factor Atf3. Tissue immunofluorescence analysis revealed that Atf3 expression was higher in the cortical osteotomy group than in the tooth movement group, and the position of Atf3 was consistent with the location of osteoclasts. To summarize, the findings indicated that corticotomy accelerated tooth movement probably by promoting the expression of Atf3 in macrophages, thus increasing their osteoblastic differentiation capacity. Our study not only elucidated the mechanism of corticotomy-accelerated tooth movement but also provided a theoretical basis for the subsequent development of noninvasive tooth movement modalities.

## 2 Methods

### 2.1 Cell treatment

RAW264.7 cells, purchased from ATCC (China), were treated with ammonium iron (III) citrate (FAC) (MedChemExpress) to simulate an environment rich in iron ions. The cells were maintained in DMEM supplemented with 10% FBS (Gibco) and 1% penicillin-streptomycin (Beyotime) Then, the cells were cultured in an incubator at 37**°**C under a humidified 5% CO_2_ atmosphere. The cells were seeded in a well plate at a density of 6.25 x 10^3^ cells/cm^2^, and after 12 h, they were treated with 40 µM FAC for four days, according to experimental requirements.

### 2.2 Animal models

In this study, 20 male SD rats (eight weeks old; weight: 200–250 g) were used. All animals were maintained in a barrier facility free from viruses and parasites and exposed to a 12-h/12-h light/dark cycle under standard conditions in the Medical Experimental Animal Center of Southwest Medical University, China. All study protocols were approved by the Committee of Southwest Medical University for Animal Resources (Ethics No. 20230705-015) and followed the ARRIVE guidelines.

The left maxillary bone was selected as the corticotomy group, and the right side as the control group in 10 rats. After anesthetizing the rats with pentobarbital, they were placed in a supine position and a 1 mm circular fixed hook was made on the upper anterior teeth using a high-speed dental handpiece. Five holes (1 mm in diameter) were made using the high-speed dental handpiece in the corticotomy group. Then a 2.0 mm orthodontic ligation wire was used to fix the closed-coil spring with constant force (50 g) between the right upper first molar and the right upper anterior tooth for tooth movement in both groups. The spring was checked daily to ensure that it had not fallen off. The rats were fed a soft diet and sacrificed 3, 7, or 14 days after the force was applied. The maxillary alveolar bones from the distal part of the second molar to the mesial surface of the first molar of both sides were collected.

### 2.3 Micro-CT analysis

The obtained samples were cleaned three times with pre-cooled PBS after removing soft tissue, and fixed with 4% polyformaldehyde for 24 h. All fixed samples underwent scanning and three-dimensional reconstruction. The alveolar bone of the first molar area was selected as the ROI area, and the BMD, Tb.Sp, and BV/TV parameters were analyzed (SkyScan1276, USA). The tooth movement distance was measured using ImageJ.

### 2.4 Tissue staining

After the bone tissue was sliced into thin sections (4 µm thick), the samples were dewaxed and rehydrated for staining.

To prepare the tissue for immunofluorescence assay, the samples were blocked with BSA for 30 min (Solarbio, China) at room temperature, followed by incubation with Atf3 (rabbit anti, Abcam, USA) and CD68 (mouse anti, BioRad, USA) (1:150) in a wet box at 4°C overnight to detect the expression of Atf3 and CD68. The following day, after rewarming and washing, the samples were incubated with Alexa Fluor^®^ 488 (goat anti-rabbit secondary antibody) and Alexa Fluor^®^ 594 (donkey anti-mouse secondary antibody) (Abcam, USA, 1:200) at room temperature for 1 h. After counterstaining the nuclei with 4′,6-diamidino-2-phenylindole (DAPI), the samples were sealed with glycerin (Solarbio, China), and the cells were observed under a fluorescence microscope.

For iron staining, the Perls staining solution was prepared following the instructions provided with the kit and incubated with the sample in a wet box at 37°C for 20 min. After washing the samples with distilled water three times, they were stained with the incubation solution at 37°C for 20 min. After washing, the enhanced working solution was added and incubated at 37°C for 20 min. The working solution was removed by washing, and then, the samples were stained with the counterstaining solution for 3–5 min. After staining, the samples were dehydrated and appeared transparent. Finally, neutral gum was used to seal the samples (Solarbio, China). The samples were observed under a microscope.

To perform TRAP staining, the samples were fixed with the TRAP fixed fluid for 1 min, and incubated with the TRAP liquid at 37°C for 50 min to stain osteoclasts, following the instructions provided with the kit (Solarbio, China). Nuclei were counterstained with hematoxylin for 3 min. After staining, the samples were dehydrated and appeared transparent. Finally, neutral gum was used to seal the samples (Solarbio, China). The samples were observed under a microscope.

### 2.5 Iron ion detection in alveolar bone

Iron ion in alveolar bone was detected using an iron assay kit (Solarbio, China), following the manufacturer’s instructions. Briefly, 50 mg of fresh alveolar bone was ground using liquid nitrogen. Then, the tissue was placed in a sterile 2 mL tube containing 1 mL of extraction solution, and then, centrifuged at 10,000 *g* (Fe^2+^) or 40,000 *g* (total iron) for 10 min. The supernatant was collected and mixed with an assay buffer to test for Fe^2+^. The total iron level was tested with the corresponding supernatant after diluting 10 times. The absorbance of the wells was measured at 593 nm (Fe^2+^) and 520 nm (total iron) using a microplate reader.

### 2.6 Transmission electron microscopy

After fixing the alveolar bone with 4% paraformaldehyde (Solarbio, China) overnight, the samples were transferred to 10% EDTA for decalcification (Solarbio, China) for three months. Then, the tissues were prepared for TEM. First, the tissues were cut into thick sections using an ultra-microtome (Leica, Germany). Then, ultrathin sections were prepared, collected on copper grids, stained with uranium acetate and lead citrate, and examined using a transmission electron microscope (JEM-1400FLASH, Japan) at 120 keV.

### 2.7 Cell isolation and single-cell RNA sequencing library construction using the 10x Genomics platform

The first and second molars and soft tissue were removed, and then, the samples were washed thrice in pre-cooled PBS containing 1% FBS. The bone tissue samples were cut into sections (1 mm thick) and digested with type I collagenase for 60 min at 37°C. Finally, the single-cell suspension was obtained by filtration through a 70-µm cell filter.

The 10x Genomics platform uses microfluidic technology to encapsulate beads and cells with a cell barcode in droplets, collect droplets containing cells, and then, lyse the cells in the droplets to connect mRNA from the cells to the cell barcode on the beads, forming single-cell GEMs. Reverse transcription was performed in the droplets to construct cDNA libraries, which differentiated the sample source of the target sequence through the sample index on the library sequence.

### 2.8 Single-cell RNA sequencing analysis

The Cell Ranger software pipeline (version 5.0.0) provided by 10×Genomics was used to demultiplex cellular barcodes, map reads to the genome and transcriptome using the STAR aligner, and down-sample reads as required to produce normalized aggregate data across samples, yielding a matrix of gene counts versus cells. The unique molecular identifier (UMI) count matrix was processed using the R package Seurat (version 4.0.0). To remove low-quality cells and multiplet captures (a major concern in microdroplet-based experiments), the following criteria were used: the cells were filtered based on (1) gene numbers (< 200), (2) UMI (< 1000), (3) log10GenesPerUMI (< 0.7), (4) proportion of UMIs mapped to mitochondrial genes (> 10%) and (5) proportion of UMIs mapped to hemoglobin genes (> 5%). Subsequently, the DoubletFinder package [2] (version 2.0.2) was used to identify potential doublets.

After applying these QC criteria, 4347 cells in the corticotomy group and 8954 cells in the control group were included in downstream analyses. The top variable genes were selected using the FindVariableGenes function. Graph-based clustering was performed to cluster cells based on their gene expression profile using the FindClusters function in Seurat. The cells were visualized using a two-dimensional Uniform Manifold Approximation and Projection (UMAP) algorithm with the RunUMAP function in Seurat. The FindAllMarkers function was used to identify marker genes of each cluster with the reference transcriptomic dataset ‘mouse.rnaseq’.

### 2.9 Differential expression analysis

Differentially expressed genes (DEGs) were selected using the FindMarkers function (test.use = presto) in Seurat. The threshold was set as P < 0.05 and |log2foldchange| > 0.58 to consider significantly differential expression. GO enrichment and KEGG pathway enrichment analysis of DEGs were performed using R based on the hypergeometric distribution. The sequencing and bioinformatics analysis were performed by OE Biotech Co., Ltd. (Shanghai, China) based on the Kyoto Encyclopedia of Genes and Genomes (KEGG) database. To identify the features of genes in the control and corticotomy groups in terms of “mineral absorption”, we used GSEA91 (http://software.broadinstitute.org/gsea/index.jsp) to assess the expression of genes in the two groups in different cell subclasses. Normalized enrichment score (NES) and false discovery rate (FDR) were used to quantify enrichment amplitude and statistical significance. Gene set variation analysis (GSVA) was performed to evaluate potential changes in pathway activity in macrophage subtypes. We used the GSVA package in the R software to perform the analysis and calculated the enrichment scores of all samples in the relevant pathways.

### 2.10 Pseudotime analysis

To investigate the origin of the differentiation of macrophages, we used the Monocle package. Based on machine learning of the expression profile of key genes, we simulated the dynamic changes of the developmental process over time. We selected the genes with a high degree of gene expression variation between cells, performed spatial dimensionality reduction based on their expression profiles, and then, constructed a minimum spanning tree (MST). Finally, using this MST, we found the longest path representing the differentiation trajectory of cells with similar transcription characteristics.

### 2.11 Transcription factor regulon analysis

To determine the changes between macrophage subtypes, we used the SCENIC software to identify modules (regulons) co-expressed by transcription factors (TFs) and potential target genes, and the regulon activity scores (RAS) of each cell. The regulon specificity scores (RSS) were used to obtain the predicted regulon and the specific relationship between each cell type. The connection specificity index (CSI) was used to represent the association between different regulons.

### 2.12 Cytotoxicity analysis

Initially, the cells were homogenously seeded in 96 well plates, and then, 6 × 10^3^ cells were added to each well to determine the effects of different concentrations of ammonium iron (III) citrate (0, 20, 40, and 80 µM) on RAW264.7 macrophages four days after induction. Cell activity assays were performed after treating the cells with the CCK‐8 kit (Apexbio), and the results were analyzed based on the absorbance measured at 450 nm using a microplate reader (Thermo Fisher Scientific).

### 2.13 Quantitative real-time PCR

Cellular RNA was extracted and reverse transcribed following the instructions provided with the RNA extraction kits (Accurate, China) and reverse transcription kits (Accurate, China). Subsequently, cDNA, primers (Sangon, China), and the SYBR green dye were placed in an octuple tube according to the instructions provided with the reagent (Accurate, China) and subjected to quantitative PCR analysis (Biorad, USA) using a predetermined program. The sequences of primers used are shown in **Table 1**.

**Table 1:** Sequences of primers.

Gene	Forward	Reverse
TNF-α	CCACCATCAAGGA CTCAA	CAGGGAAGAATCTGG AAAGG
IL1β	GCCACCTTTTGACAG TGATGAG	ATGTGCTGCTGCGAG ATTTG
IL-4	AACGAGGTCACAGGA GAAGG	TGGAAGCCCTACAGA CAAGC
IL-6	TAGTCCTTCCTACCCCA ATTTCC	TTGGTCCTTAGCCAC TCCTTC
IL-10	CCAGTACAGCCGGG AAGACA	GAAGGCAGTCCGCA GCTCTA
CD86	ACGGAGTCAATGAAGA TTTCCT	GATTCGGCTTCTTGTG ACATAC
CD206	CTTCGGGCCTTTGGA ATAAT	TAGAAGAGCCCTTGG GTTGA
GAPDH	GTCTTC ACCACCATGGAG	CCAAAGTTGTCATGGA TGACC

### 2.14 Flow cytometry

The cells to be processed were placed in flow cytometry tubes and counted to ensure that each tube contained 10^6^ cells. Staining buffer was added to wash the cells, which were centrifuged at 300 g for 5 min. After discarding the supernatant, Fc blocker was added, followed by incubation at 4°C in the dark for 30 min. After sealing, the samples were washed twice with the staining buffer and centrifuged at 300 g for 5 min. Then, the samples were mixed with F4/80 and incubated with the CD86 antibody mixture at 4°C in the dark for 30 min. The samples were washed twice with the staining buffer and centrifuged at 300 g for 5 min. Next, Fix/Perm solution was added to resuspend the cells for membrane breaking, and then, the cells were incubated at 4°C in the dark for 20 min. The cells were washed with Perm/Ash Buffer twice and centrifuged at 300 g for 5 min, after which, the supernatant was discarded and CD206 was added. The samples were washed at 4°C in the dark for 30 min, washed twice with Perm/Flash Buffer, centrifuged at 300 g for 5 min, and then, 300–350 µL of the staining buffer was added to each tube to resuspend cells. The cells were filtered with a 70 µm filter and analyzed using a flow cytometer. Finally, the results were analyzed using the Novoexpress software.

### 2.15 Statistical analysis

All measurements were performed at least three times. All data were presented as the mean ± standard deviation (SD). For normally distributed data, the differences between the two groups were determined by conducting Student’s t-tests, and differences among multiple groups were determined by ANOVA followed by *post hoc* Bonferroni correction. The homoscedasticity of the data was examined by conducting Bartlett’s test and the Brown-Forsythe test. Non-homoscedastic data were analyzed by the Brown-Forsythe ANOVA test followed by Dunnett’s T3 multiple comparisons test. All results were considered to be statistically significant at P < 0.05, and all analyses were performed using Prism (version 8; GraphPad).

## 3 Results

### 3.1 Corticotomy reduces local mineralization and accelerates tooth movement

**Figure 1:**
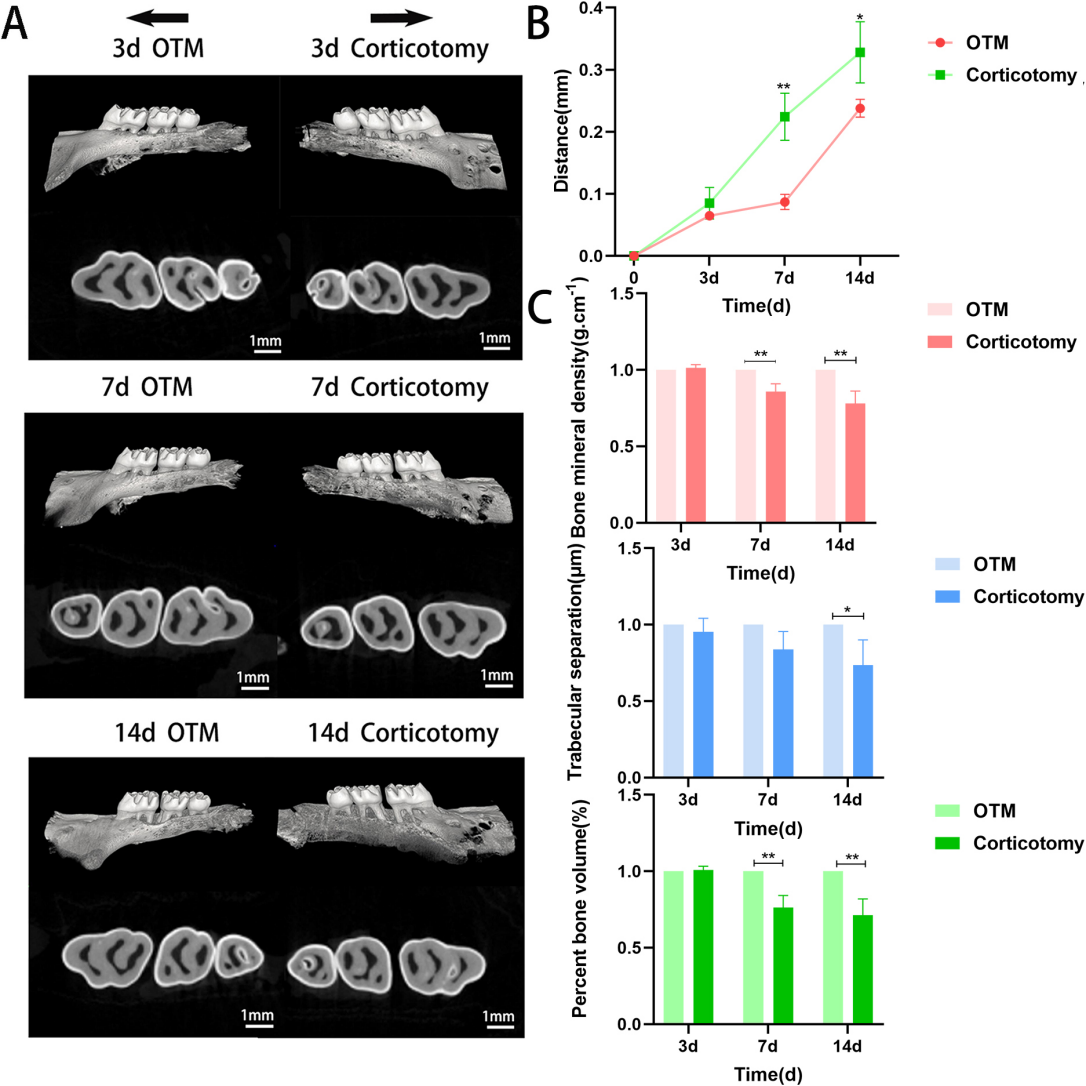
Corticotomy reduces local mineralization and accelerates tooth movement **(A)** Three-dimensional reconstruction of the maxillary alveolar bone (black arrows indicate the direction of tooth movement); **(B)** Changes in tooth movement distance; **(C)** Bone parameter analysis (BMD, BV/TV, and Tb.Sp). * indicates P < 0.05, ** indicates P < 0.01, the OTM group.

To simulate corticotomy-accelerated tooth movement, we used high-speed handpieces and mechanical force devices to mimic this model. We measured the movement distance of bilateral maxillary first molars and changes in bone indices on days 3, 7, and 14 by micro-CT examination (**Figure 1A**). Tooth movement was faster in the corticotomy group than in the OTM group over time (**Figure 1B**), but local alveolar mineralization, trabecular thickness, bone separation, and buccal bone plate thickness were lower (**Figure 1C**). These results indicated that the changes in bone, caused by corticotomy, may directly accelerate tooth movement.

### 3.2 Single-cell sequencing reveals tissue iron accumulation in alveolar bone

**Figure 2:**
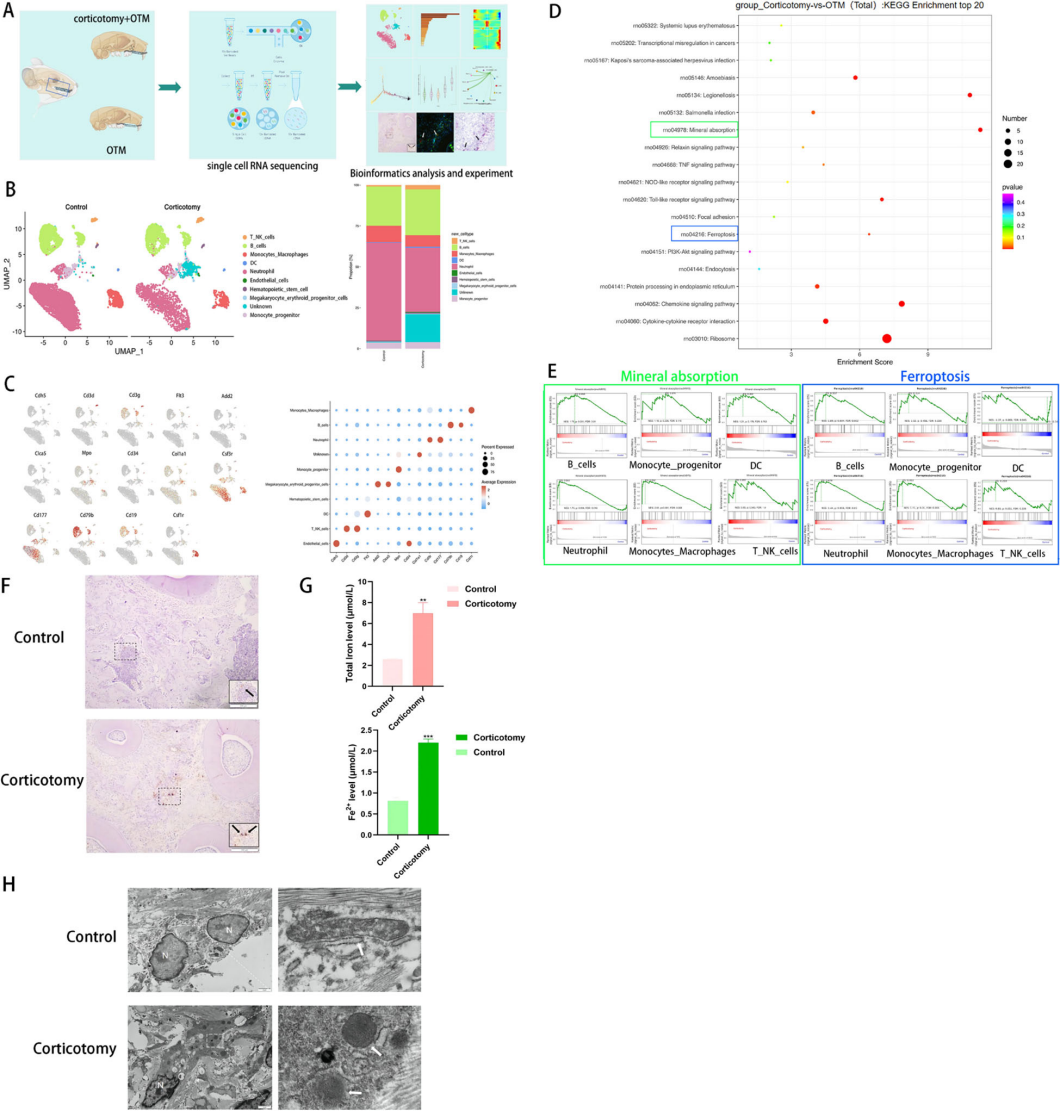
Single-cell sequencing reveals tissue iron accumulation of alveolar bone **(A)** A diagrammatic representation of the study design. Bilateral maxillary alveolar bone was used for scRNA-seq with 10x Genomics. The following integrated analysis of single-cell transcriptome data is described in squares. **(B)** UMAP plots of 10,628 cells from the OTM group and 5,861 cells from the corticotomy group (n = 3 SD rats) are presented, showing 10 clusters in each plot. Each cluster is presented in a different color; **(C)** Expression levels of selected known marker genes are illustrated in the UMAP plots, and feature plots of the control and corticotomy groups are shown. **(D)** KEGG enrichment analysis. **(E)** GSEA score of each cell type for “mineral absorption” and “ferroptosis”. **(F)** Iron staining of tissue sections (black arrow indicates iron). **(G)** Fe^2+^ and total iron expression level. **(H)** Changes in mitochondrial structure using TEM (N indicates the nucleus; white arrows indicate mitochondria). ** indicates P < 0.01, and *** P < 0. 001 compared to the control group.

To elucidate the mechanisms underlying corticotomy-assisted tooth movement, we isolated cells from bilateral alveolar bones of SD rats (**Figure 2A**). After performing quality control using Cell Ranger (10x Genomics), we obtained 10,628 cells from the control group and 5,861 cells from the corticotomy group for subsequent analysis. The number of cells indicated local cell death induced by corticotomy (**Supplementary Figures 1A, B**). A dimensional reduction clustering analysis of these cells was conducted, relying on PCA for dimensionality reduction, UMAP for visualization, and SNN for optimal cell clustering (**Supplementary Figure 2A**). However, marker gene expression and function in cluster 6 were not clear (**Supplementary Figures 2B, C**, **3A, B**), suggesting the presence of cell debris near the quality control threshold (**Supplementary Figure 2C**); thus, we classified it as unknown. Based on the results of the clustering analysis, the cells were divided into eight cell types, according to the specific marker gene expression, including neutrophils, B cells, DCs, monocyte progenitors, monocyte-macrophages, T-NK cells, endothelial cells, and hematopoietic cells, among others (**Figure 2B**), categorized using markers such as Csf3r, CD177, CD79b, CD19, Flt3, Mpo, Csf1r, CD3g, CD3d, Cdh5, Col1A1, and CD34 (**Figure 2C**).

The results of functional enrichment analysis suggested that corticotomy exacerbates local inflammatory responses and affects cellular mineral absorption and iron death processes (**Figure 2D**). GSEA scoring for “mineral absorption” and “ferroptosis” processes showed the highest enrichment in monocyte-macrophage cells (**Figure 2E**). Tissue iron staining and iron levels confirmed that iron accumulation was higher in the corticotomy group (**Figures 2F, G**). The mitochondrial ridge decreased or disappeared and the outer mitochondrial membrane (OMM) ruptured in the corticotomy group (**Figure 2H**).

### 3.3 Single-cell map shows changes in macrophage subtypes after corticotomy

**Figure 3:**
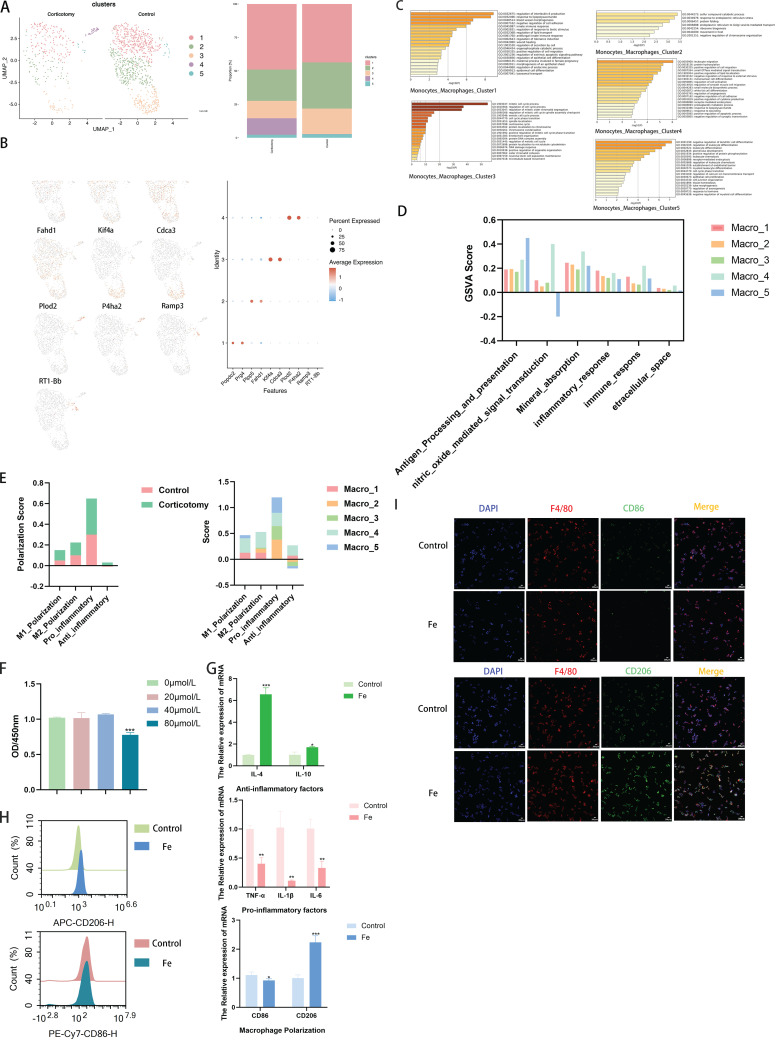
The single-cell map shows changes in macrophage subtypes after corticotomy **(A)** The UMAP plots of monocytes_macrophages from the OTM group and the corticotomy group (n = 3 SD rats) illustrate five clusters in each plot. **(B)** Expression levels of selected known marker genes are illustrated in UMAP plots and feature plots from the control and corticotomy groups in monocytes_macrophages. **(C)** Metascape of each cluster in monocytes_macrophages. **(D)** GSVA of each cluster of monocytes_macrophages. **(E)** The expression of macrophage polarization markers and inflammatory factors of each cluster in monocytes_macrophages. **(F)** Cytotoxicity of different CoCl_2_ concentrations (0 μM, 50 μM, 100 μM, 200 μM, and 400 μM) on RAW264.7 cells on day 4 (*** indicates *P < 0.001* compared to the 0 μM group); **(G)** Expression of the mRNAs of TNF-α, IL-1β, IL-4, IL-6, IL-10, CD86, CD206, and GAPDH was detected via PCR assays (* indicates *P < 0.05*, ** indicates *P < 0.01*, and *** *P < 0. 001* compared to the control group); **(H)** Macrophage polarization was detected by flow cytometry; **(I)** Expression of CD86 and CD206 was detected by immunofluorescence.

As iron metabolism in macrophages was highly enriched, we analyzed changes in macrophage subtypes. Dimensional reduction clustering analysis revealed five macrophage subtypes, indicated by genes such as Popdc2, Prg4, Plpp5, Fahd1, Kif4a, Cdca3, Plod2, P4ha2, Ramp3, and RT1-Bb (**Figures 3A, B**). The results of enrichment analysis revealed the functions of each subtype using Metascape, including regulation of interleukin-6 production, blood vessel morphogenesis, cell cycle processes, leukocyte migration, and dendritic cell differentiation (**Figure 3C**). Gene set scoring of “antigen presentation”, “extracellular space”, “immune response”, “inflammatory response”, “mineral absorption”, and “inhibition of cell proliferation” in each subtype indicated that cluster 4 played a key role in immune and inflammatory responses and significantly affected mineral absorption (**Figure 3D**). Moreover, analysis of macrophage polarization markers and inflammatory factors showed that corticotomy increased the polarization of M1 and M2 macrophages, with stronger anti-inflammatory and pro-inflammatory capacities, especially in cluster 4. Cluster 4 showed stronger anti-inflammatory abilities (**Figure 3E**). Additionally, our results showed no toxicity below 40 μM after ammonium iron (III) citrate induction (**Figure 3F**). RAW264.7 macrophages polarized to the M2 type in the iron ion environment *in vitro* (**Figures 3G, I**). Iron inhibited the production of pro-inflammatory factors and promoted the generation of anti-inflammatory factors (**Figure 3H**).

### 3.4 Atf3 regulates a new direction for osteoclast differentiation

**Figure 4:**
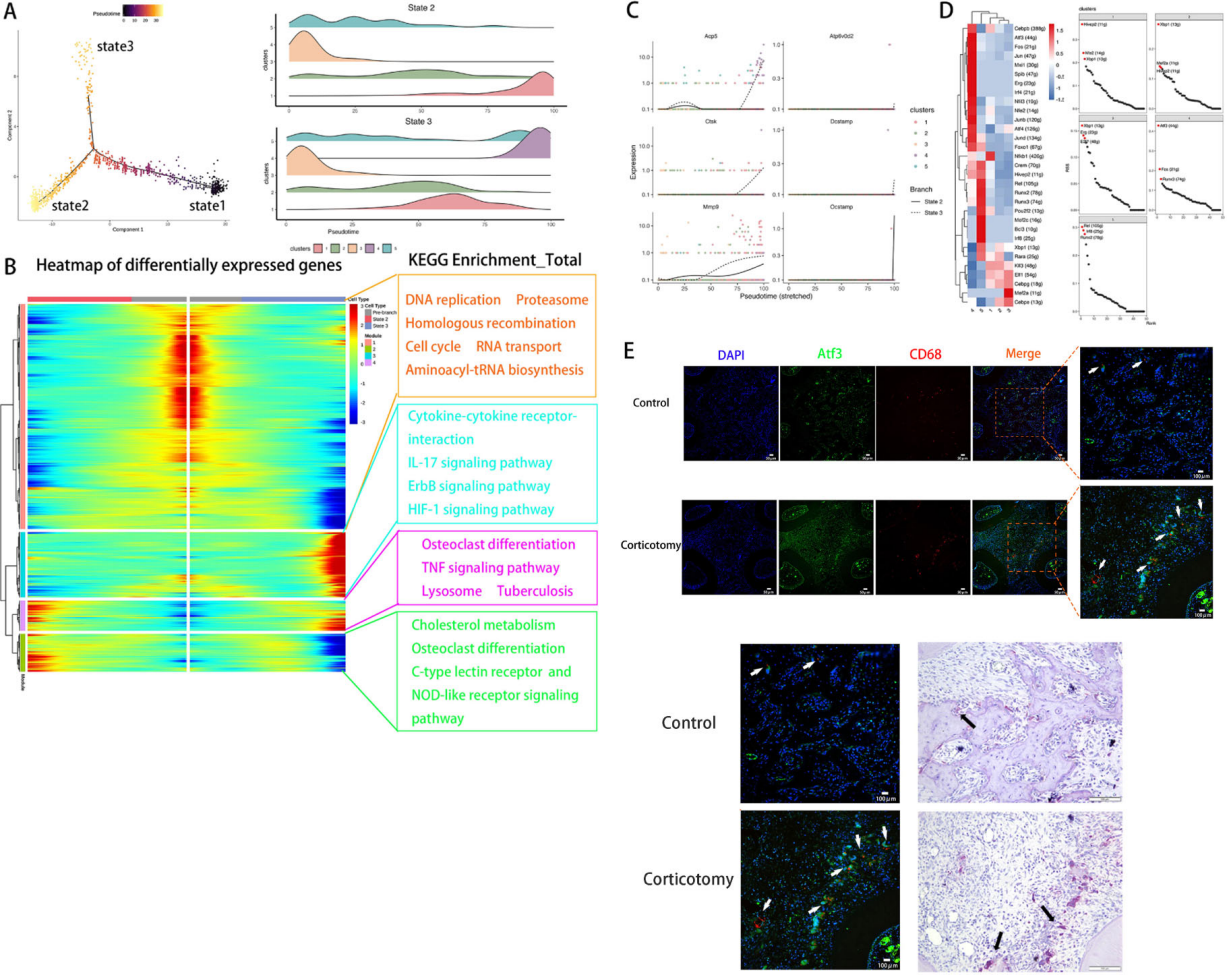
Pseudotime and SCENIC analyses revealed that Atf3 regulates a new direction for osteoclast differentiation **(A)** The monocytes_macrophages developmental trajectory was determined using Monocle. **(B)** A heat map of DEGs was made based on their simple kinetics through pseudotime using Dynverse and KEGG enrichment of each module. **(C)** The pseudotime expression trend diagram of osteoclast-related genes (Acp5, Ctsk, Mmp9, Dcstamp, Ocstamp, and Atp6v0d2) using pseudotime analysis; **(D)** A heat map of differentially expressed regulons in each monocyte_macrophage and the regulon specificity ranking diagram of each monocyte_macrophage. **(E)** ICC of CD68 and Atf3 and TRAP staining of osteoclasts in the control and corticotomy groups (white arrow indicates Atf3 and black arrow indicates the corresponding osteoclast).

Based on changes in genes, we defined three states using Monocle2. We defined state 1 as pre-branch (**Figure 4A**). By comparing the developmental trajectories of macrophages between the two groups, we found that state 3 is a unique developmental direction after corticotomy (**Figure 4A**). Additionally, to analyze the functional changes in different clusters during the developmental process, we re-clustered genes with similar expression patterns into four modules in the developmental trajectory (**Figure 4B**). We found that module 3, which is specifically expressed in state 3, is primarily related to inflammatory pathways such as the “IL-17 pathway”, “Cytokine-cytokine receptor interaction”, and the “ErbB signaling pathway” (**Figure 4B**). This suggests that state 3 represents a direction of differentiation in response to trauma stimulation. By analyzing the developmental trajectory of osteoclast-related markers, we found that markers related to osteoclastic differentiation in cluster 4 were mainly highly expressed in the late developmental stage (**Figure 4C**). To further identify the key TFs regulating the transformation of macrophage subtypes, we conducted a SCENIC analysis to assess the key genes. Our results suggested that the TF activities of cluster 4 were the highest among all macrophage subtypes, with Atf3 being the most prominent one (**Figure 4D**). The results of immunofluorescence assays showed that the expression of CD68 and Atf3 increased in the corticotomy group. Moreover, the position of Atf3 was consistent with the position of osteoclasts (**Figure 4E**), indicating that Atf3 might be a key cell affecting the transformation of macrophages into osteoclasts.

### 3.5 Cell communication reveals changes in cell communication after corticotomy

**Figure 5:**
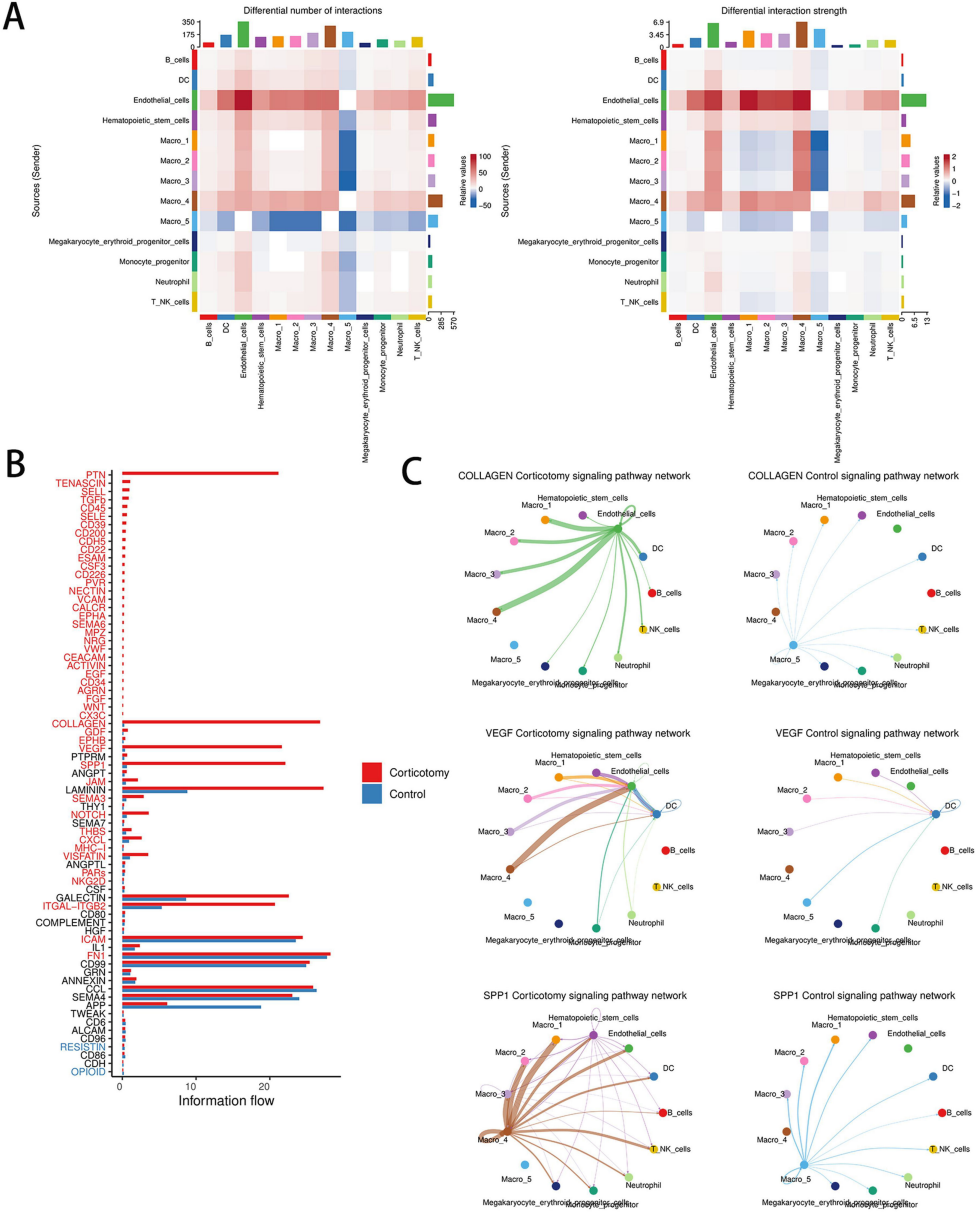
Corticotomy can change cell communication patterns **(A)** A heat map of the number and intensity of intercellular interactions between the control group and the corticotomy group is shown. **(B)** A diagram of the overall information flow for each signal pathway between the control group and the corticotomy group is presented. **(C)** The communication network diagram of the COLLAGEN signaling pathway, VEGF signaling pathway, and SPP1 signaling pathway in the control and corticotomy groups is illustrated.

To understand the interactions between cells, we evaluated cell communication. The results showed that the outgoing and incoming signals between endothelial cells and cluster 4 macrophages were significantly enhanced (**Figure 5A**). Among the 73 signaling pathways examined, the strength of the interactions in the COLLAGEN, VEGF, and SPP1 pathways showed the most notable changes post-corticotomy (**Figure 5B**). The analysis of receptor-ligand pairs showed that Col4a1-CD44 contributed primarily to the COLLAGEN signaling pathway, VEGF-a-VEGFR1 to the VEGF signaling pathway, and SPP1-CD44 to the SPP1 signaling pathway (**Figure 5C**). An increase in the expression of CD44 suggested that the interaction between endothelial cells and cluster 4 macrophages through COLLAGEN and SPP1 might influence the regulation of immune responses after corticotomy. Moreover, the VEGFa-VEGFR1 signaling pathway, a classic signal promoting angiogenesis, was primarily mediated by cluster 4 macrophages after corticotomy. These results suggested that the new state of macrophages not only plays a key role in accelerating tooth movement after corticotomy but also participates in the subsequent repair process.

## 4 Discussion

In this study, by conducting micro-CT examinations, we found that corticotomy reduces local bone mineralization. The trauma caused by corticotomy triggers local bone remodeling, leading to the acceleration of tooth movement.

In the enrichment analysis, we found that “mineral absorption” and “ferroptosis” were highly enriched in the corticotomy group, especially in macrophage subgroups. Ferroptosis is a form of cell death that depends on iron (14) and is triggered by iron overload and lipid peroxidation (15). It plays an important role in inflammation, cancer, and injury (16). In macrophages, the inflammatory environment and RANK-induced classical macrophage osteoclastic differentiation can trigger ferroptosis (17, 18). We also detected iron overload and ferroptosis after corticotomy, along with an increase in osteoclasts. This is the first report of ferroptosis in osteoclasts in a corticotomy model.

Based on the dimensionality reduction analysis of macrophages, we discovered a new macrophage development trajectory in the corticotomy group, mainly consisting of a new macrophage subcluster, known as cluster 4. This subcluster exhibits prominent functions in “immunity”, “inflammation”, “mineral absorption”,” ferroptosis”, and “osteoclast differentiation”. The SCENIC analysis revealed that the TF Atf3 is the key driving gene of this cell subtype. By staining tissues, we found that the number of local macrophages significantly increased after corticotomy, and Atf3 was mainly expressed in macrophages and highly coincided with the position of TRAP staining positive cells. Interestingly, Atf3-deficient mice did not show abnormal bone mass under normal conditions, but under inflammatory conditions, osteoclast differentiation was significantly weakened (19). *In vitro* studies have also shown that Atf3 deficiency in macrophages can reduce osteoclast differentiation induced by RANKL (20).

Some studies have shown that Atf3 production is mainly related to endoplasmic reticulum (ER) stress, cytokines, chemokines, and LPS induction (21). In diseases such as osteoporosis and osteoarthritis, Atf3 expression is upregulated (22, 23). When Atf3 expression is eliminated, symptoms of arthritis decrease considerably (24). These findings indicate that in bone tissue, Atf3 plays a key role in response to various stimuli, such as inflammation, trauma, and mechanical stress, and initiates bone remodeling.

Regarding cell communication, we found that after corticotomy, the signal strength between endothelial cells and cluster 4 of macrophages increased significantly and the COLLAGEN, VEGF, and SPP1 signaling pathways were the most active. Previous studies have shown significant interactions between endothelial cells and macrophages. After an injury, endothelial cells may promote macrophage recruitment through the COLLAGEN signaling pathway to regulate inflammatory responses. We found similar results in this study. Moreover, cluster 4 can regulate endothelial cells to participate in angiogenesis through the VEGF pathway, indicating that in response to the traumatic stress of corticotomy, macrophages not only differentiate to induce bone absorption but also participate in the subsequent repair process. We found that under normal conditions, Atf3 can promote the polarization of macrophages toward the M2 type (25). By comparing the polarization of macrophages, we found that the polarization of M1-type and M2-type macrophages increased after corticotomy, and the transformation of M2-type macrophages in cluster 4 was more prominent. These findings suggested that cluster 4 may also participate in the anti-inflammatory process, which might also explain the time-limited problem of corticotomy. After activating the repair process, due to the loss of external stimulation, macrophages expressing high levels of Atf3 can no longer increase their osteoclast differentiation ability and exhibit more anti-inflammatory effects.

To summarize, our study revealed that corticotomy induces ferroptosis, especially in macrophages. Atf3 may play a key role in the response of macrophages to the stimulation of corticotomy and the development of a new developmental trajectory. The altered macrophages may not only participate in osteoclast differentiation but also have anti-inflammatory and angiogenesis-promoting effects. These findings reveal a theoretical basis for the mechanism of corticotomy-accelerated tooth movement. As a key target, Atf3 may facilitate non-invasive accelerated tooth movement through the development of related targeted drugs. Additionally, targeting and inhibiting Atf3 may block the progression of bone-destructive diseases such as osteoarthritis.

This study had some limitations. Although the experiments revealed changes in transcription at the single-cell level, the spatial location of the cells was not determined. Therefore, future studies need to implement spatial transcriptomic sequencing to obtain more information. This study only assessed the preliminary effect of changes in iron on macrophage polarization and osteoclast differentiation after cortical osteotomy. However, the precise mechanism underlying the regulatory effects of iron on macrophage phenotype needs to be elucidated. Additionally, further experiments are needed to determine the regulation of iron metabolism by Atf3 in osteoclasts in macrophages.

## Data Availability

The data presented in the study are deposited in the NCBI online repositories, accession number "SRR29757196; SRR29757197
