# Supplementary material for: Single-cell sequencing decodes the secrets of the RAP phenomenon of corticotomy
Source: Front Immunol. 2024 Oct 1;15:1397727. doi: 10.3389/fimmu.2024.1397727 (PMC11487196; doi:10.3389/fimmu.2024.1397727)
Supplement: Supplementary file 1 [file DataSheet1.docx]

**Single Cell Sequencing Decodes the Secrets of RAP phenomenon of Corticotomy**

Z.B. Fan ^1,2^, S.H. Li ^1,2^, L.P. You ^1,2^_，_Y.X. Lan ^1,2^, Y.T ZHONG ^1,2^, Y.F. Ma ^1,2^, J. Xu ^1,2^, X.X. Xu^1,2^

**Appendix Figure**

**
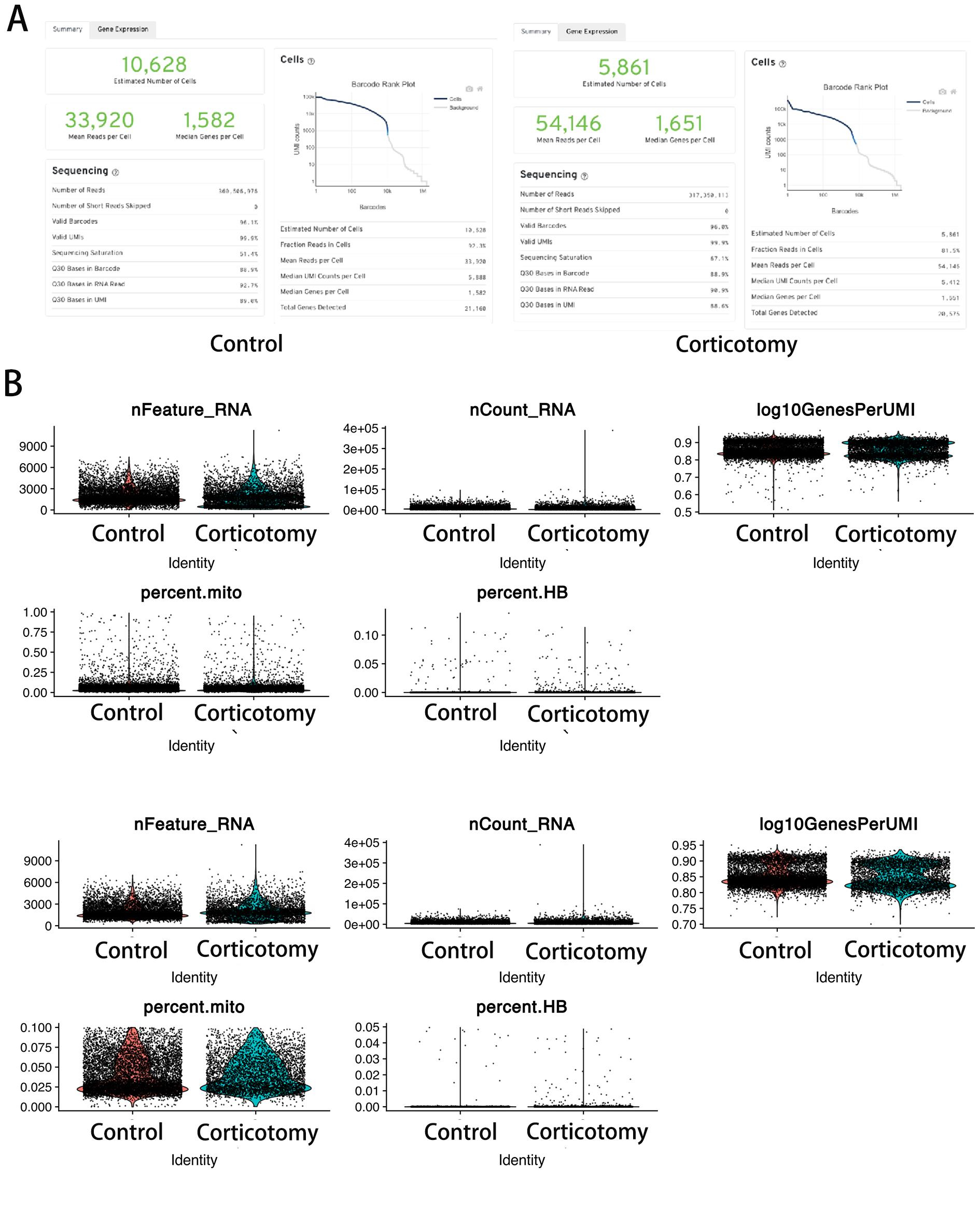
**

**Appendix Figure 1 Raw data quality assessment and cell ranger of control and corticotomy group**

1. Raw data quality assessment; B. Quality control of control group and corticotomy group.

**
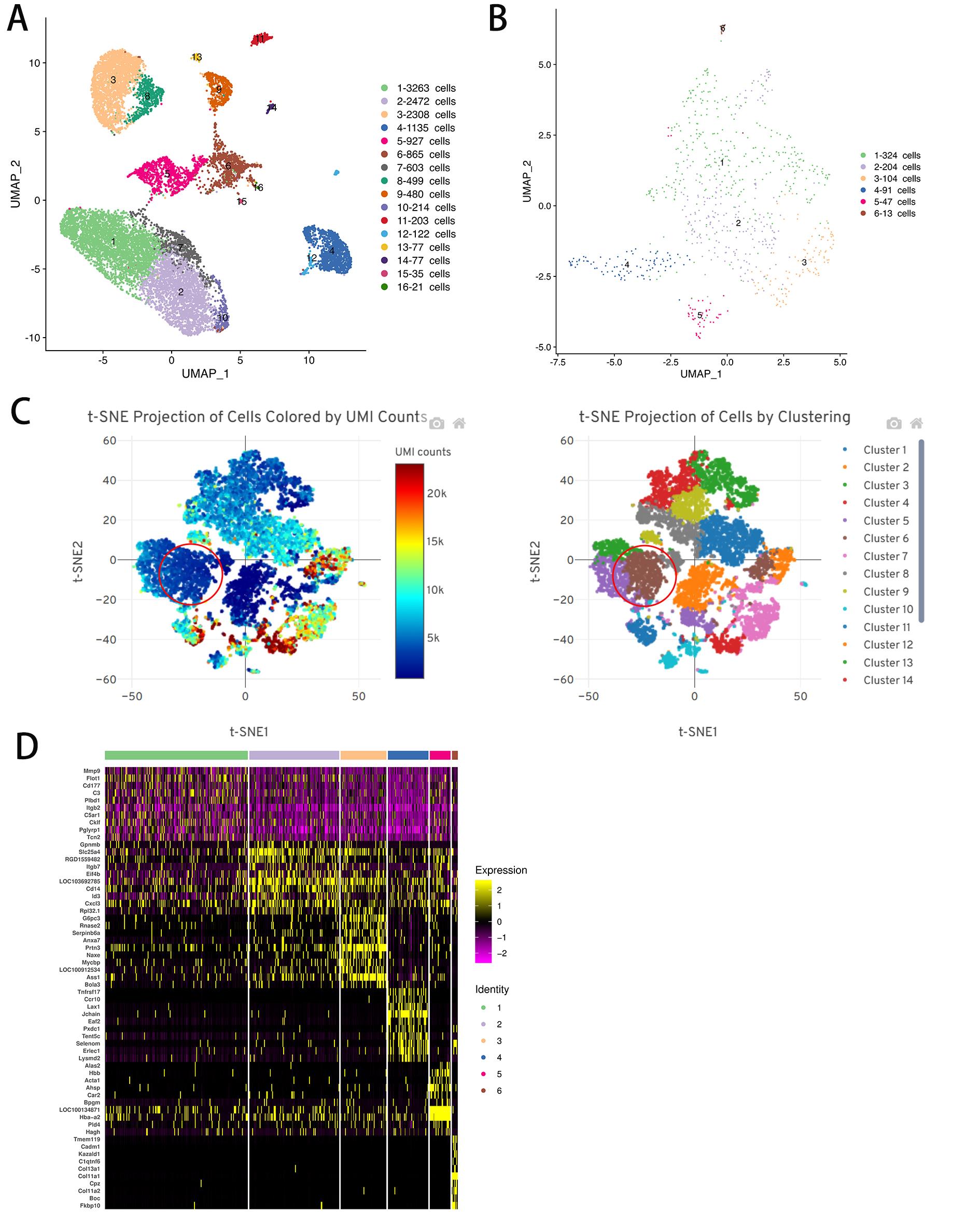
**

**Appendix Figure 2 Cell heterogeneity analysis**

1. UMAP plots of control group and corticotomy group; B. UMAP plots of cluster6 control group and corticotomy group; C. Heatmap of marker genes of cluster6; D. t-SNE Projection of Cells Colored by UMI Counts and clustering.


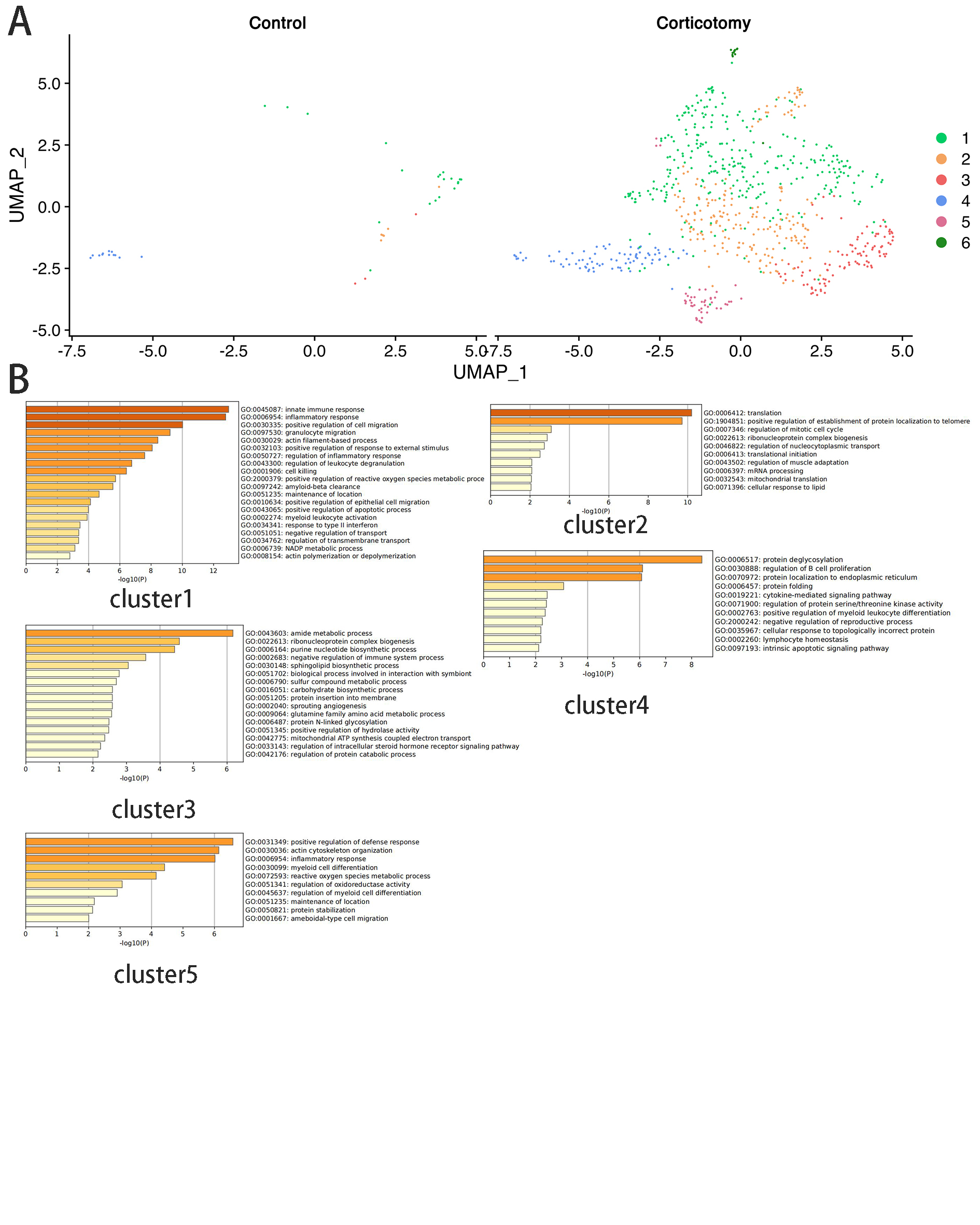


**Appendix Figure 3 Function analysis of “unknown”**

A.The UMAP plots of “unknown” from the OTM group and the corticotomy group；B. Metascape of each cluster in “unknown”。
